# Expansion of Human Mesenchymal Stromal Cells from Fresh Bone Marrow in a 3D Scaffold-Based System under Direct Perfusion

**DOI:** 10.1371/journal.pone.0102359

**Published:** 2014-07-14

**Authors:** Adam Papadimitropoulos, Elia Piccinini, Sophie Brachat, Alessandra Braccini, David Wendt, Andrea Barbero, Carsten Jacobi, Ivan Martin

**Affiliations:** 1 Departments of Surgery and of Biomedicine, Institute for Surgical Research and Hospital Management, University Hospital Basel, University of Basel, Basel, Switzerland; 2 MusculoSkeletal Diseases, Novartis Institutes for Biomedical Research, Basel, Switzerland; French Blood Institute, France

## Abstract

Mesenchymal stromal/stem cell (MSC) expansion in conventional monolayer culture on plastic dishes (2D) leads to progressive loss of functionality and thus challenges fundamental studies on the physiology of skeletal progenitors, as well as translational applications for cellular therapy and molecular medicine. Here we demonstrate that 2D MSC expansion can be entirely bypassed by culturing freshly isolated bone marrow nucleated cells within 3D porous scaffolds in a perfusion-based bioreactor system. The 3D-perfusion system generated a stromal tissue that could be enzymatically treated to yield CD45- MSC. As compared to 2D-expanded MSC (control), those derived from 3D-perfusion culture after the same time (3 weeks) or a similar extent of proliferation (7–8 doublings) better maintained their progenitor properties, as assessed by a 4.3-fold higher clonogenicity and the superior differentiation capacity towards all typical mesenchymal lineages. Transcriptomic analysis of MSC from 5 donors validated the robustness of the process and indicated a reduced inter-donor variability and a significant upregulation of multipotency-related gene clusters following 3D-perfusion- as compared to 2D-expansion. Interestingly, the differences in functionality and transcriptomics between MSC expanded in 2D or under 3D-perfusion were only partially captured by cytofluorimetric analysis using conventional surface markers. The described system offers a multidisciplinary approach to study how factors of a 3D engineered niche regulate MSC function and, by streamlining conventional labor-intensive processes, is prone to automation and scalability within closed bioreactor systems.

## Introduction

MSC are receiving an increasing experimental and clinical interest, owing to the large degree of plasticity and the capacity to modulate the immune system or the phenotype of cancer cells [1]. Their use is thus advocated for treatment of various genetic, haematologic or immunologic pathologies and in the emerging field of regenerative medicine [2]–[4]. For most of these potential applications, given the low frequency among bone marrow nucleated cells (around 0.01%), MSC are typically expanded by sequential passages in monolayer (2D) cultures. However, this process is associated with a progressive reduction of their clonogenicity and multilineage differentiation capacity, and is often accompanied by cellular senescence [5], [6].

Studies on different cellular systems have led to the concept that maintenance of ‘early progenitor’ properties generally requires a tissue-specific microenvironment or niche [7]–[11], which can hardly be resembled by the plastic substrate and 2D configuration of tissue culture flasks [12]. Various attempts have thus been reported to expand MSC in three-dimensional (3D) environments, based on suspension culture in the presence of dynamic flow [13], [14], on microcarrier beads [15]–[17] or on a rotating bed bioreactor system [18], [19]. Despite the promising results obtained, however, these approaches required an initial phase of MSC growth on plastic, which is intrinsically associated with the selection of the adherent cellular fractions, possibly already depleted of the less adherent earlier progenitors [20], and the loss of most hematopoietic lineage cells. Indeed, non-mesenchymal bone marrow cells were proposed to be involved in regulating MSC function [21] and have been demonstrated to enhance growth of MSC with clonogenic properties [22], [23].

We previously reported that the continuous perfusion of freshly isolated human bone marrow cells directly through the pores of 3D ceramic-based scaffolds resulted in the reproducible generation of tissue constructs, which were highly osteogenic upon ectopic implantation in nude mice [24]. By eliminating the 2D culture step, the system not only streamlined the MSC culture process, but also supported the maintenance of hematopoietic lineage cells, including some of the early progenitors (i.e., CFU-GEMM), thereby establishing some features of the bone marrow niche [25].

In this study, we aimed at investigating the use of the above described 3D scaffold-based perfusion system for human MSC expansion. For this purpose, the generated constructs were enzymatically processed and the retrieved cells were phenotypically and functionally compared to those generated following conventional expansion protocols. Furthermore, a microarray analysis was introduced to identify potential new molecular markers and pathways differentially regulated as well as to validate the robustness of the process across different donor preparations.

## Materials and Methods

### Bone Marrow Aspirates

Bone marrow aspirates (20 ml volumes) were obtained from five healthy donors (average age 45 y.o.) after informed consent during orthopaedic surgical procedures in accordance with the local ethical committee (University Hospital Basel; Prof. Dr. Kummer; approval date 26/03/2007 Ref Number 78/07). Nucleated cells were isolated from aspirates by means of red blood cells lyses buffer (pH 7.2) containing 0.15 M NH_4_CL, 1 mM KHCO_3_ (both from Sigma, Switzerland) and 0.1 mM Na_2_EDTA (Fluka, Switzerland). The average clonogenicity (number of fibroblast colony-forming units; CFU-f) in the fresh marrow aspirates was 0.008%±0.002%.

### Culture Medium

Unless otherwise stated, complete medium (CM) consisted of α-Modified Eagle's Medium supplemented with 10% fetal bovine serum (FBS), 10 mM HEPES buffer, 1 mM sodium pyruvate, 10000 U/ml penicillin and 10000 µg/ml streptomycin (all from GIBCO, Switzerland). CM was then supplemented with 10 nM dexamethasone and 0.1 mM L-ascorbic acid-2-phosphate (both from Sigma, Switzerland) and with 5 ng/ml fibroblast growth factor-2 (FGF-2, R&D systems, Europe).

### MSC Culture

Using a perfusion bioreactor system described in [26] and now commercially available by Cellec Biotek AG (http://www.cellecbiotek.com), an average of 66×10^6^ freshly isolated bone marrow–nucleated cells were perfused for 5 days through 8-mm-diameter, 4-mm-thick disks of porous (total porosity, 83%±3%; accessible surface area 3200 cm^2^) hydroxyapatite ceramic (Engipore; Fin-Ceramica Faenza, Faenza, Italy, http://www.finceramicafaenza.com) at a superficial velocity of 400 µm per second. After 5 days, culture medium was replaced and perfusion culture was performed at a velocity of 100 µm per second for additional 14 days and changing the medium twice per week. In order to establish a comparison with the standard culture process, MSC expansion in 2D (in 56 cm^2^ Petri dishes; BD Biosciences) was performed for up to 19 days without passaging using similar initial cell numbers/surface area and schedule of medium changes, as in the 3D expansion condition.

### Cell Extraction

At the end of the expansion phase in the 3D culture system, cells were extracted by substituting the CM with a solution of 0.3% collagenase (collagenase) and perfusing the ceramic constructs for 40 min followed by 0.05% trypsin/0.53 mM EDTA solution (trypsin) for additional 15 min both at 400 µm per second. Extracted cells were subsequently sorted using anti-CD45-coated magnetic beads (Miltenyi Biotec, Auburn, CA), according to the manufacturer's instructions. 2D-expanded cells were retrieved by using the same enzymatic solutions, i.e. collagenase for 40 min and trypsin for 5 min. The fraction of dead cells, preliminarily assessed by assessed by Trypan blue exclusion (Sigma, Switzerland), was negligible (less than 3%), with no obvious differences between the experimental groups. Both CD45^+^ and CD45^−^ viable cell populations were assessed for the ability to form fibroblastic colonies. The CD45^−^ populations were further characterized by flow cytometry, gene expression by means of microarray analysis and quantitative real-time (QRT) PCR or tested for the multilineage differentiation capacity, as described below.

### Clonogenicity (CFU-f) and flowcytometry assays

CFU-f assays (n = 5) of bone marrow or expanded cells were performed by plating 4400 freshly isolated mononucleated cells or 4 expanded cells per cm^2^ in tissue culture dishes, respectively. The procedure was optimized following preliminary experiments with serial dilutions of plated cells. After 14 days of culture, cells were fixed in 4% formalin, stained with 1% methylene blue and the number of colonies was counted.

2D or 3D-perfusion expanded CD45^−^ cells from one donor were incubated with antibodies against CD29, CD31, CD34, CD44, CD45, CD49a, CD73, CD90, CD105, CD117, CD133, CD144, CD146, CD166, CD271, Alkaline phosphatase, SSEA-1 or human leucocyte antigen (HLA)-DR (all from BD Biosciences). Isotype IgGs were used as controls (all from BD Biosciences). After washing, cells were suspended in FACS buffer (0.5% human serum albumin, 0.5 mm EDTA in PBS) and analysed with a FACSCalibur flow cytometer (BD Biosciences).

### RNA Extraction and Microarray analysis

Total cellular RNA (40 ug) was extracted from 2D or 3D-perfusion expanded CD45- cells, obtained from 5 independent experiments/donors, using RNeasy Micro kit (Qiagen, Valencia, CA) following the protocol supplied by the manufacturer. RNA were hybridized to Affymetrix Human HG-U133plus2 GeneChip arrays according to the manufacturer recommendations. All the data have been deposited in the Gene Expression Omnibus database with experiment series number GSE52896 available at http://www.ncbi.nlm.nih.gov/geo/query/acc.cgi?acc=GSE52896.

Arrays pre-processing and analysis were performed using R and the Bioconductor package (http://www.bioconductor.org/) and passed through array quality control using the AffyQCreport tool. Raw intensities were normalized using RMA and scaled to a 2% trimmed mean of 150. Probes with normalized expression values below 50 in both groups were filtered out. Differential gene expression was performed using Limma. Probes were annotated using the platform annotation file version 31 from NetAffx. Genes with a fold change higher than 2 and an adjusted P-value below 0.05 (Benjamini and Hochberg multiple testing correction) were considered regulated. Data from microarrays were analysed by Principal Component Analysis (PCA) using TM4 Multi Experiment Viewer (MeV), available at http://www.tigr.org/software/tm4/mev.html in order to ascribe the overall variability of the sample to a limited number of variables.

To validate microarray data, the expression of a set of genes was evaluated by quantitative real-time (QRT) PCR (Figure S2). Total RNA extraction, cDNA synthesis and real-time reverse transcriptase-polymerase chain reaction (RT-PCR; 7300 AB Applied Biosystem) were performed to quantitate expression levels of the following genes of interest: CXCL12 (CXCL12-Applied Biosystems, Ref. Number: Hs00171022_m1), STC1 (STC1- Applied Biosystems, Ref. Number: Hs00174970_m1), EDNRB (EDNRB-Applied Biosystems, Ref. Number: Hs00240747_m1), FZD5 (FZD5-Applied Biosystems, Ref. Number: Hs00258278_s1), CXCL5 (CXCL5-Applied Biosystems, Ref. Number: Hs01099660_g1), KYNU (KYNU-Applied Biosystems, Ref. Number: Hs01114099_m1), CCL20 (CCL20-Applied Biosystems, Ref. Number: Hs01011368_m1), TAC1 (TAC1-Applied Biosystems, Ref. Number: Hs00243225_m1), DNER (DNER-Applied Biosystems, Ref. Number: Hs01039911_m1), EREG (EREG-Applied Biosystems, Ref. Number: Hs00914313_m1), NR4A3 (NR4A3-Applied Biosystems, Ref. Number: Hs00545009_g1), SLC6A15 (SLC6A15-Applied Biosystems, Ref. Number: Hs00375196_m1) and SNF1LK (SNF1LK-Applied Biosystems, Ref. Number: Hs00545020_m1). 18s was used as housekeeping (18s-Applied Biosystems, Ref. Number: Hs03003631_g1).

### Bionformatic Analysis

#### Gene Set Enrichment Analysis (GSEA)

The list of regulated genes was ranked according to the relative fold-change and loaded in GSEA software (http://www.broadinstitute.org/gsea/index.jsp; ver. 2.0.12). A variety of genesets from the Molecular Signatures Database (MSigDB) were analyzed (http://www.pnas.org/cgi/content/abstract/102/43/15545). The list of genes related to osteogenic differentiation was based on the Human Osteogenesis RT^2^ Profiler PCR Array (SABiosciences).

### Database for Annotation, Visualization and Integrated Discovery (DAVID) and Cytoscape

Functional enrichment analysis for up- and down-regulated genes (2 fold with an adjusted pvalue below 10^−2^) was performed using the open-source web-based DAVID platform (http://david.abcc.ncifcrf.gov/) including Gene Ontology (GO) and Pathways categories. Enriched functional categories and pathways were clustered by gene overlap using Enrichment Map in Cytoscape [27], [28] and labelled for recurrent keywords using the WordCloud plugin (http://baderlab.org/Software/WordCloudPlugin). In the generated Cytoscape diagram, the node size is proportional to the number of genes defining the node. Edges connect nodes that share common genes and edge thickness is proportional to the number of shared genes between nodes.

### Multilineage differentiation assays

The osteogenic differentiation capacity was tested by culturing cells, obtained from 3 independent experiments/donors, for 2 weeks in CM further supplemented with 100 nM dexamethasone, 10 mM β-glycerophosphate, and 0.05 mM ascorbic acid-2-phosphate. After 2 weeks, cell layers were either stained with alizarin red solution to evidence mineral deposition or assessed for ALP activity normalized to cell numbers, as previously described [29]. Shorter culture time with respect to the commonly used in literature 3 weeks protocol was chosen in order to maximize the differences regarding the *in vitro* osteogenic differentiation capacity of MSC between the two experimental conditions.

The adipogenic differentiation capacity was tested by alternating cycles of cell culture with different media, including 10 µg/ml insulin, 10 µM dexamethasone, 100 µM indomethacin, and 500 µM 3-isobutyl-1-methyl xanthine (adipogenic induction medium) or 10 µg/ml insulin (adipogenic maintenance medium) as previously described [30]. After a total of 14 days, the presence of adipocytes was microscopically documented and quantified following Oil red-O staining.

The chondrogenic differentiation capacity was tested by culturing cells in spherical pellets, formed by gentle centrifugation in 1.5 ml conical polypropylene tubes (Sarstedt, Numbrecht, Germany), in serum-free D-MEM medium (GIBCO, Switzerland) containing TS^+^1 (10 µg/ml insulin, 5.5 µg/ml transferrin, 5 ng/ml selenium, 0.5 mg/ml bovine serum albumin, 4.7 µg/ml linoleic acid; Sigma, Switzerland), 0.1 mM ascorbic acid 2-phosphate, 1.25 mg/ml human serum albumin, 100 nM dexamethasone (Sigma, Switzerland), and 10 ng/ml TGF-β1 (R&D Systems, Europe), with medium changed twice weekly. After 3 weeks' culture, pellets were processed biochemically for glycosaminoglycan (GAG) and DNA content and histologically for Safranin-O staining.

### Immunosuppression assay

The proliferation of CD4+ T cells, sorted from PBMCs of a healthy donor, in the presence of MSC was performed in 96-well plates following a method described [31]. Briefly, 2D- and 3D-perfusion expanded MSC were seeded at densities of 1250, 5000 and 20000 cells per well and allowed to attach at least 4 h at 37oC with RPMI1640 medium supplemented with 10% FBS, 10 mM HEPES buffer, 1 mM sodium pyruvate, 10000 U/ml penicillin and 10000 µg/ml streptomycin (all from GIBCO, Switzerland), before adding 100000 CD4+ cells in the presence of 1 ug/ml of the mitogen phytohemagglutinin (PHA; Remel Europe Ltd. Clipper Boulevard West, Crossways Dartford,Kent, DA26PT UK). After 56 h of co-culture, 1 µCi/well ^3^H-thymidine (GE Healthcare, Little Chalfont, United Kingdom) was added to each well and incubated for additional 16 h. Cells were then harvested and the ^3^H cpm counted by a scintillation beta-counter to measure the radioactivity in DNA recovered from the cells in order to determine the extent of cell division. Each condition was tested in triplicate.

### Statistical analysis

For the microarray analysis, genes with a fold change higher than 2 and an adjusted P-value below 0.05 (Benjamini and Hochberg multiple testing correction) were considered regulated. Results are reported as mean ± SD. Statistical analysis was performed with GraphPad Prism 4.0 (Graph Pad software, La Jolla, CA, USA). Differences were assessed using Mann–Whitney U-tests and considered statistically significant with P<0.05.

## Results

### 3D-perfusion expansion of freshly isolated MSC

**Figure 1:**
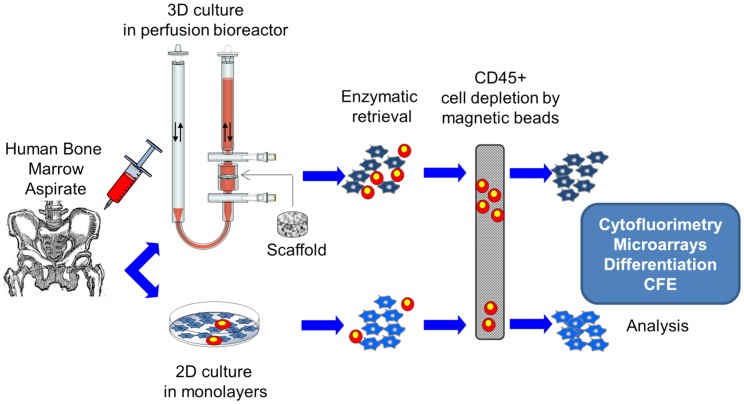
Schematic overview of the experimental setup. Bone marrow aspirates were seeded into the 3D perfusion system and in conventional Petri dishes. After culture, cells from both systems were enzymatically retrieved and CD45- sorted cells using magnetic beads were analyzed as described.

**Figure 2:**
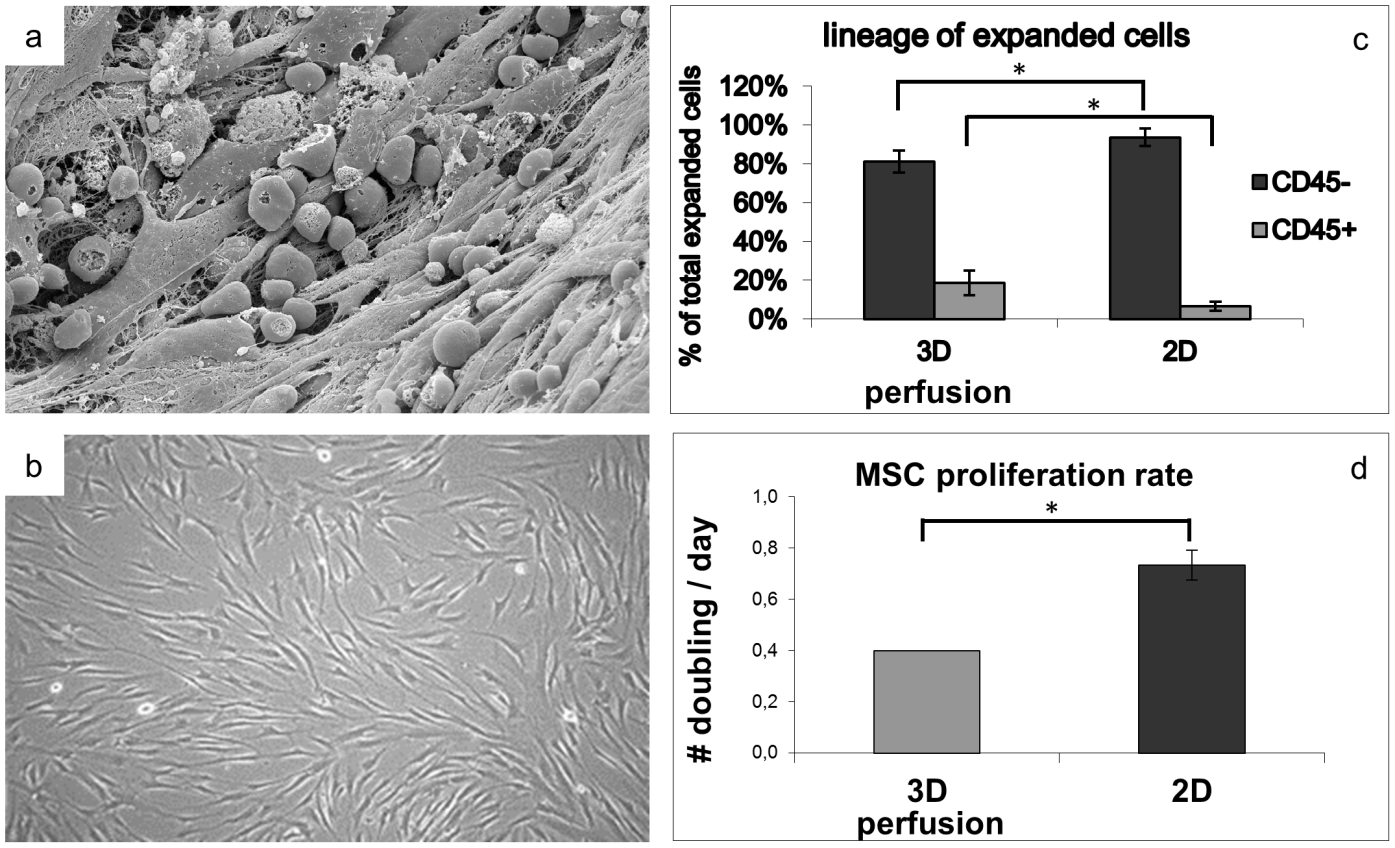
Phenotypical and growth characteristics for 2D and 3D perfused MSC. (a) Scanning electron microscopy imaging of cells within the scaffold display a complex network of branched fibroblastic-like adherent cells and the presence of rounded cells possibly of hematopoietic origin. (b) 2D cultured MSC display a typical flat fibroblastic morphology. (c) Flow cytometry of cultured cells shows a higher frequency of CD45+ cells in the perfusion system. (d) Proliferation rates indicate higher proliferation in 2D as compared to 3D perfusion cultured MSC. Statistically significant differences (P<0.05) are indicated with an asterisk (*; n = 5).

Using a bioreactor system as described in [26] and graphically illustrated in Figure 1, total BM cells were perfused through the scaffold pores for 5 days (cell seeding phase), followed by perfusion of culture medium for further 14 days (cell culture phase). Based on the retrospectively calculated density of CFU-f from the five donors (0.08%±0.02%) and assuming that all CFU-f attached to the ceramic scaffolds, an estimated average of 5.4×10^3^MSC were perfused through each scaffold, corresponding to 1.6 MSC per cm^2^ of ceramic surface area. This process resulted in the formation of stromal-like tissue structures, including cells of heterogeneous morphologies in physical contact with each other (Figure 2a). Instead, conventional cell culture in Petri dishes using similar cell density per surface area led to the generation of adherent cells, typical of the fibroblastic phenotype (Figure 2b). Enzymatic retrieval of the cells from both conditions and labelling for CD45 indicated the presence of a significantly higher percentage of cells of the hematopoietic lineage after expansion in 3D-perfusion as compared to 2D (19.3±5.7% vs 6.0±4.5% CD45+) (Figure 2c). The extent of MSC proliferation in the 3D perfusion system, assuming that all harvested CD45- cells (total of 1.36+/−0.34×10^6^ cells/scaffold) were of the mesenchymal lineage and derived from the initial relative number of seeded CFU-f, retrospectively estimated by clonogenicity assays, was of 7.6±1.7 doublings, corresponding to about 0.4 doublings/day. MSC growth in plastic dishes within the same time frame of 19 days (total of 1.76+/−0.46×10^6^ cells/dish) was significantly higher, corresponding to 0.74 doublings/day (Figure 2d). Based on the measured cell yields, the same numbers of cells occupying 56 cm^2^ of a 10 cm diameter Petri dish could be expanded in ∼0.2 cm^3^ of scaffold volume.

### Phenotypic characterisation of 3D-perfusion expanded MSC

**Figure 3:**
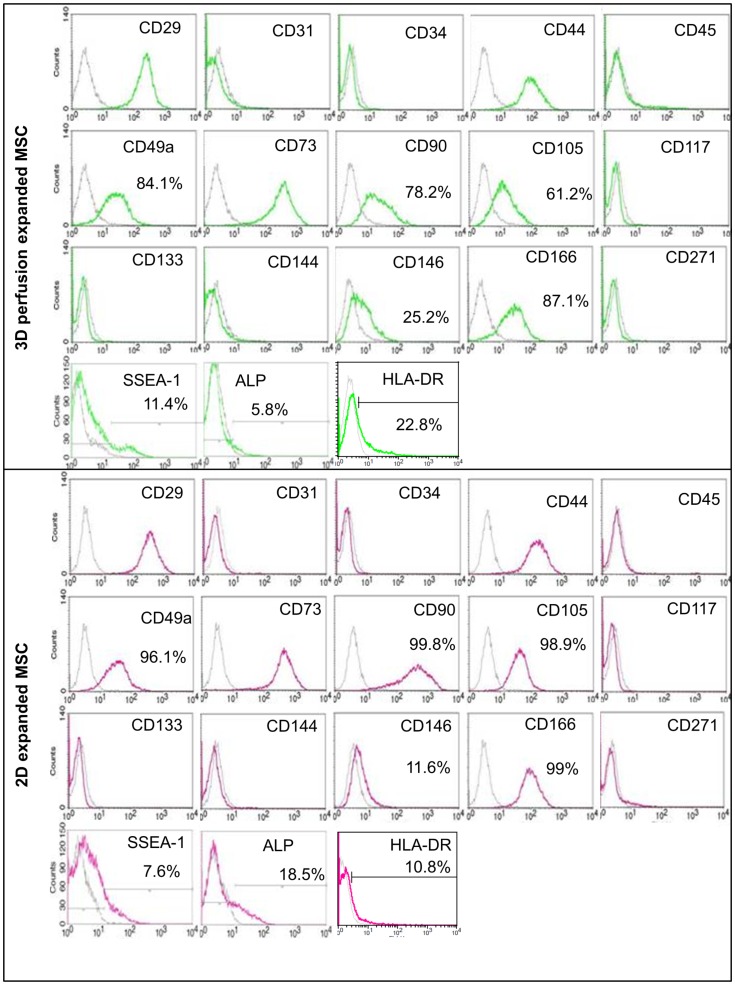
Analysis of the expression of surface markers in 2D and 3D cultured MSC. Colored lines display the frequency of positive cells compared to isotype (gray lines). Most markers were similarly expressed in the two experimental groups. CD90, CD105, CD166, and ALP positive populations were more represented in monolayer culture, while CD146 and SSEA-1 were more represented in 3D-perfusion culture.

In order to investigate the phenotype of the mesenchymal cells, retrieved cells were negatively sorted for the expression of CD45. Results displayed in Figure 3 indicate a large overlap in the cytofluorimetric profile of the two cell-expanded groups, without clear-cut differences in the presence or absence of specific cell populations. However, as compared to 2D-expansion culture, a lower percentage of MSC expanded by 3D-perfusion expressed CD90 78.2% vs 99.8%), CD105 (61.2% vs 98.9%), CD166 (87.1 vs 99%) and ALP (5.8% vs 18.5%), a marker associated with the osteoblastic differentiation of MSC. Moreover, slightly higher percentage of 3D-perfusion expanded MSC were positive for HLA-DR (22.8% vs 10.8%) or for CD146 (25.2% vs 11.6%) and SSEA-1 (11.4% vs 7.6%), which were proposed to be associated with progenitor cell properties [32]–[37].

### Microarray analysis of 2D- and 3D-perfusion expanded MSC

**Figure 4:**
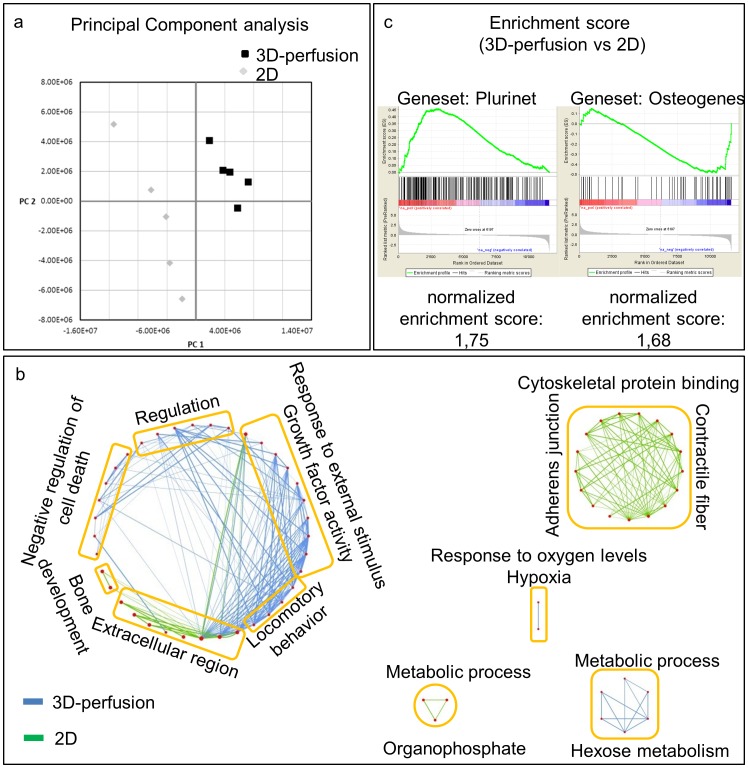
Gene expression analysis of MSC cultured in 2D or 3D perfusion culture. (a) Principal component analysis on global gene expression data. Cells cultured in 3D perfusion system exhibited a significantly different RNA expression profile compared to 2D, with lower inter-donor variability. (b) Cytoscape diagram integrates graphically the most relevant gene ontology biological processes identified by functional annotation (DAVID bioinformatics tool) of regulated genes. Node size (red dots) is proportional to the number of genes defining the node. Edges connect nodes that share common genes in the 2D condition (green edges) or in the 3D perfusion condition (blue edges). Edges thickness is proportional to the number of shared genes between nodes. (c) Gene set enrichment analysis of regulated ranked genes displays that up-regulated genes in 3D perfusion condition are largely overlapping with stem cell related genes (Geneset: Plurinet), while gene related to osteogenic differentiation (Geneset: Osteogenes) are down-regulated. The acronym for NES stands for normalized enrichment score, which is calculated by GSEA software.

In order to broaden the search of potential differentially expressed markers and to validate the robustness of the process across different donor preparations, the CD45- fractions of bone marrow cells expanded by 3D-perfusion or in 2D from 5 independent donors were profiled using expression microarrays. Exploratory analysis using PCA (data dimensionality reduction) was performed in order to reveal correlations between the samples [38]. By dot-plotting the data derived by the two experimental groups, it is possible to estimate the similarity between each sample as a function of the distance of each pair of dots. This analysis shows a striking separation of the samples from 2D and 3D-perfusion on Principal Component 1 (PC1), confirming that culture conditions represented the most influential factor in discerning among cell preparations (Figure 4a). Interestingly, samples derived from cells cultured in 2D were more spread along the PC2 axis as compared to 3D-perfusion ones, suggesting a higher inter-donor variability induced by 2D-expansion. After pre-processing, we identified 702 genes (343 up-regulated and 359 down regulated) with a fold change of 2 and an adjusted p-value of 10^−2^. A list of the 10 more up- and down-regulated genes is reported in Table 1.

**Table 1:** Top-ten of significant up- and down-regulated expressed genes.

Probe Set ID	Gene Descirption	Gene Symbol	Function	Fold Change (3D-perfusion vs 2D)
**Upregulated**				
214974_x_at	chemokine (C-X-C motif) ligand 5	CXCL5	Secreted	69
205239_at	amphiregulin	AREG	Both	46
205476_at	chemokine (C-C motif) ligand 20	CCL20	Secreted	39
230748_at	solute carrier family 16, member 6 (monocarboxylic acid transporter 7)	SLC16A6	Tm	32
226281_at	delta/notch-like EGF repeat containing	DNER	Tm	27
206336_at	chemokine (C-X-C motif) ligand 6 (granulocyte chemotactic protein 2)	CXCL6	Secreted	26
204105_s_at	neuronal cell adhesion molecule	NRCAM	Tm	24
206376_at	solute carrier family 6 (neutral amino acid transporter), member 15	SLC6A15	Tm	23
211506_s_at	interleukin 8	IL8	Secreted	21
205767_at	epiregulin	EREG	Both	20
**Downregulated**				
230204_at	hyaluronan and proteoglycan link protein 1	HAPLN1	Secreted	−28
204051_s_at	secreted frizzled-related protein 4	SFRP4	Secreted	−18
212328_at	LIM and calponin homology domains 1	LIMCH1		−17
227662_at	synaptopodin 2	SYNPO2		−16
225275_at	EGF-like repeats and discoidin I-like domains 3	EDIL3	Secreted	−16
228407_at	signal peptide, CUB domain, EGF-like 3	SCUBE3	Secreted	−16
220976_s_at	keratin associated protein 1-1	KRTAP1-1		−15
223315_at	netrin 4	NTN4	Both	−14
212327_at	LIM and calponin homology domains 1	LIMCH1		−14
212865_s_at	collagen, type XIV, alpha 1	COL14A1	Secreted	−13

In order to investigate the pathways associated with the 2D versus the 3D cultures, we performed a Gene Ontology (GO) enrichment and cluster analysis using the online web-platform DAVID on the derived list of regulated genes. The statistically enriched pathways can be visualized as an enrichment map with nodes being pathways and edges representing the overlap in genes in these pathways (Figure 4c). The main GO categories increased in 3D-perfusion vs 2D-expanded MSC were the “Monosaccharides metabolic processes (fructose and glucose)”, “Chemokine activity”, “Inflammatory response”, “Response to hypoxia” and “Negative regulation of apoptosis” (Table S1). Consistent with the multicellular tissue-like morphology observed during 3D-perfusion expansion, the GO functional categories “Positive regulation of multicellular organismal process” and “Extracellular space” were also significantly over-represented in the list of up-regulated genes in 3D culture. Conversely, GO categories related to “Fat-related (phospholipid and sphingolipid) and organophosphate metabolic processes” as well as to cytoskeleton, contraction and adhesion were found to be decreased in the 3D-perfusion vs 2D-expanded MSC (Table S2). Figure 4b displays a representation of the resulting GO categories and the identified pathways from Tables S1 and S2, linked to the underlying biological processes. Interestingly, “Bone development” was found to be up-regulated in the 2D-expanded cells and appeared to be consistent with the increased protein expression of ALP. Furthermore, one of the most significant 3D up-regulated geneset uncovered using GSEA analysis is the PluriNet [39], a matrix of global gene expression profiles of various types of stem cells, supporting the more “stem cell” like transcriptional footprint of our 3D-perfusion model. Also the geneset describing osteoblastic differentiation was down-regulated in the 3D-perfusion vs the 2D-expanded MSC (Figure 4c).

### Validation of the in vitro functionality of 3D-perfusion expanded MSC

**Figure 5:**
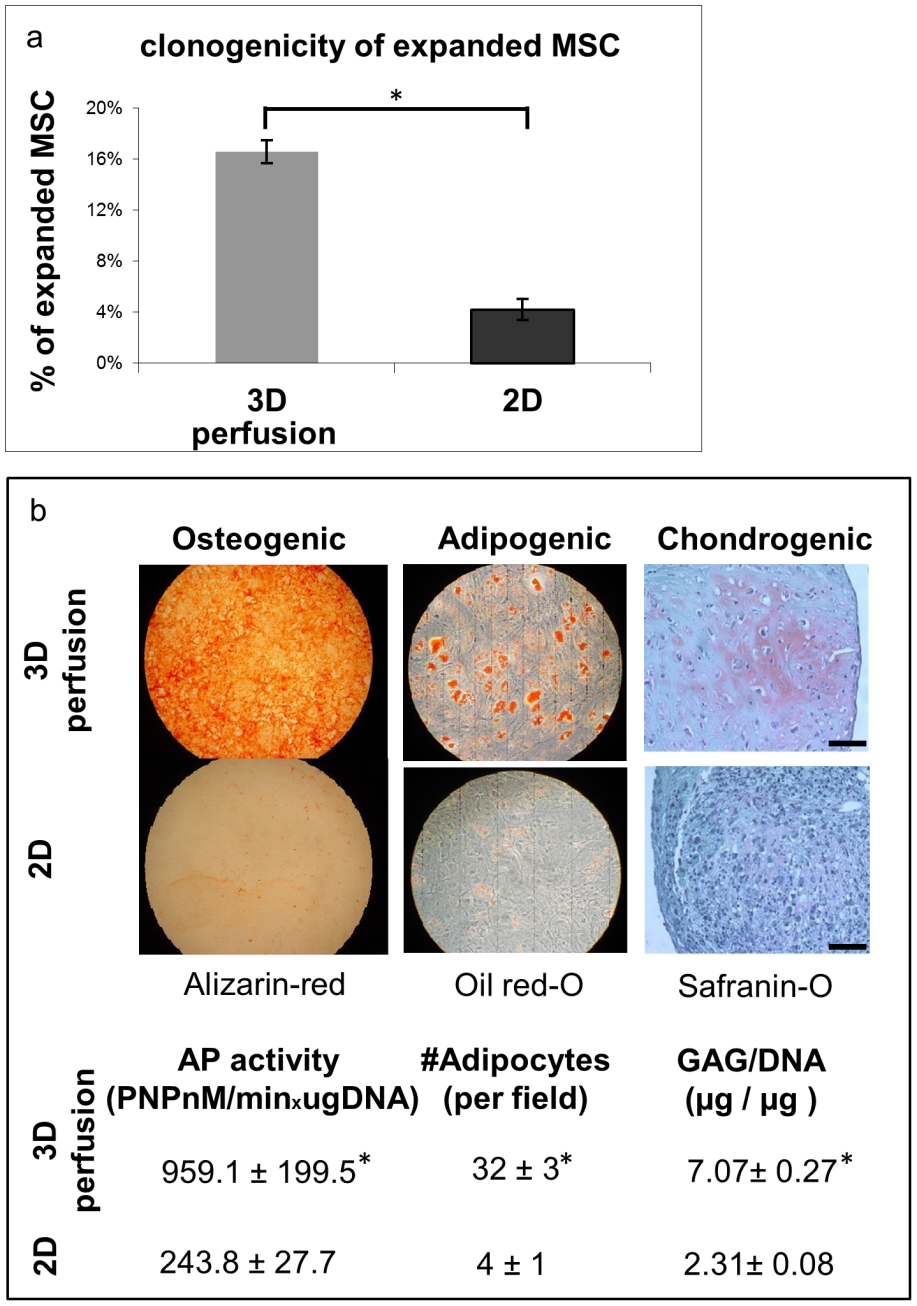
Functional differences between 2D and 3D perfused MSC. Higher (a) frequency of clonogenic cells and (b) differentiation capacity for osteogenic, adipogenic, and chondrogenic lineages with the associated quantifications of 3D perfusion- as compared to 2D-expanded cells. Scale bar: 50 um. Statistically significant differences (P<0.05) are indicated with an asterisk (*; n = 3).

We next investigated whether the differential gene expression accounting for multi-potency maintenance and differentiation was mirrored in the functionality of CD45- cells expanded by 3D-perfusion or 2D. The CD45- fraction of the 3D perfusion-cultured cells included a 4.3-fold higher percentage of clonogenic cells (Figure 5a) than that of cells expanded on plastic for the same time (respectively 17% vs 4%), suggesting a better preservation of progenitor cell features. This hypothesis was further confirmed by a more efficient multi-lineage differentiation capacity upon exposure to typical chondrogenic, osteogenic and adipogenic conditions, as determined by histochemical and quantitative biochemical assays (Figure 5b). Since the number of doublings by MSC expanded for 19 days under 3D-perfusion or 2D was different, cell populations were compared also using a shorter culture time in 2D (i.e., 14 days), leading to 9.1 total doublings and thus more similar to the 3D-perfusion group. Both the clonogenic cell fraction and the multilineage differentiation profile of the shorter expansion time in 2D (data not shown) were comparable to those determined for the longer expansion time. Notably, both 2D- and 3D-perfusion expanded MSC cells shared similar anti-proliferating effects on activated CD4+ cells when co-cultured in vitro (Figure S1). Lastly and as expected, CD45+ cells from both experimental groups did not contain adherent fibroblastic clonogenic cells when re-plated in Petri dishes, confirming efficient magnetic depletion (data not shown).

## Discussion

We have developed a system for the expansion of MSC which entirely bypasses the use of 2D surfaces by seeding and expanding fresh bone marrow preparations directly within the pores of 3D scaffolds under perfusion flow. As compared to the conventional 2D culture system, MSC expanded under 3D-perfusion (i) preserved better their early progenitor properties, as they maintained a higher clonogenicity and a superior multilineage differentiation capacity, (ii) did not lose their anti-proliferative function, based on a standard in vitro assay typically used to claim ‘immunomodulation’ properties, and (iii) displayed reduced inter-donor variability and consistent upregulation of multipotency-related pathways, as assessed by transcriptomic analysis.

Identifying a strategy for efficient expansion of MSC preserving their functionality is a critical target towards fundamental mechanistic studies on their biological properties, as well as for their prospective clinical use in the field of tissue engineering and regenerative medicine [12]. Among several hurdles, the absence of phenotypic markers that uniquely identify populations of MSC with specific functions challenges the definition of a quality control during MSC culture [40]. Indeed, surface proteins typically used to characterize MSC [41] were not differentially expressed in cells expanded in 2D or by 3D-perfusion, indicating that they are not suitable to capture functional features related to superior clonogenicity and multilineage differentiation. Only a limited set of markers, including CD146 and SSEA-1, were expressed by a larger percentage of 3D-perfusion expanded MSC, consistent with the proposed association of those markers to earlier progenitor/stem populations of MSC [34]–[37]. HLA-DR was found to be expressed in both conditions, likely due to the presence of FGF-2 in the culture medium [42], [43]. The relatively higher HLA-DR expression observed in 3D-perfusion condition is consistent with previous observations on the effect of hematopoietic cells on MSC [43] and did not alter the anti-proliferative effects of MSC on T-cells. The broader impact of HLA-DR expression on the immunomodulatory properties of MSC is still subject of debate.

A genome wide comparison demonstrated a clear separation between the transcriptomes of MSC expanded in 2D or 3D-perfusion as evidenced by PCA; moreover, a reduced dispersion of 3D-perfused samples indicates that culture conditions can diminish the inter-donor variability that typically affects 2D cultures. Gene set enrichment analysis further demonstrated that following expansion under 3D-perfusion, MSC up-regulated or maintained a transcriptome profile similar to that of other stem cells, supporting the superior maintenance of the experimentally verified MSC multipotency. In this context, after the expansion phase bone related pathways were found down-regulated in the 3D-perfusion group, further indicating better preservation of an undifferentiated MSC phenotype, against the default progression towards the osteoblastic lineage [44]. Consistently, epidermal growth factor like ligands, which were highly upregulated in the 3D-perfusion dataset, were previously shown to be important for maintenance of osteoprogenitor cells at an undifferentiated stage [45]. Following the differentiation induction phase, 2D-expanded cells, displayed a limited osteogenic profile, despite their apparently more “osteoblastic” phenotype after expansion. Although an in vivo test of osteogenicity was not performed, the in vitro data seem to indicate that the spontaneous tendency to express osteoblastic genes does not necessarily reflect a superior efficiency of functional differentiation.

Amongst the highest differentially upregulated genes found in the 3D-perfusion dataset, several ones coded for toll-like receptors (TLR), interleukins (IL) and other chemokines which are known to be involved in processes of cell migration, tissue homeostasis and repair, as well as in the regulation of immunologic responses. In particular, the higher expression of TLR-2 together with IL-6 and IL-8 may indicate the activation of the receptor by its associated ligands, which has been previously proposed to regulate MSC multipotency [46]. The established 3D transcriptional profiles described here highlighted differential expression of several transmembrane related genes, which may represent a starting point for future studies to define novel markers for the prospective isolation of earlier MSC progenitors.

Some of the categories identified from GO enrichment analysis during 3D-perfusion expansion were related to hypoxia, negative regulation of apoptosis and cell metabolism. Previous studies reported the positive role of hypoxia, a physiological feature of the niche of MSC [47], on the cell maintenance in an undifferentiated state, with metabolic features associated with an extended and more genetically stable lifespan [48]. In the described 3D-perfusion culture system, oxygen gradients and thus hypoxic regions may have occurred as a result of the relatively low rate of fluid flow passing through compact areas of cell-laid ECM. Future studies will have to further explore the role of hypoxia by either using smaller sized scaffolds, thereby enhancing oxygen transport, or by performing 2D cultures in hypoxic conditions.

For 2D expanded cells, cytoskeletal binding, contractile fiber and adherence junction pathways were up-regulated. These biomechanical ECM-induced processes were previously reported to influence cell fate [49], [50] and induce osteogenesis of MSC, independently from the culture conditions [51]. Indeed, it has been shown that MSC sense the stiffness of their environment through physical contact and contraction of ECM proteins, which are deposited according to the rigidity of the underlying material surface [50]. Here, the up-regulation of these processes in 2D expanded MSC may be possibly explained by their continuous exposure to the rigid surface of plastic, in contrast to the 3D-perfusion system, where cells were progressively embedded within ECM (Figure 2), of most likely lower stiffness.

The two experimental conditions for MSC expansion differed in multiple parameters of various nature (e.g., 3D vs 2D configuration, ceramic vs plastic substrate, flow-induced shear vs static environment, maintenance vs loss of hematopoietic cells), which can hardly be de-coupled to establish appropriate controls. Thus, while confirming the influence of a dynamic 3D environment on MSC properties [52], the identification of the mechanisms leading to a more functional population of MSC when expanded under 3D-perfusion is beyond the scope of the present work. It is likely, however, that the 3D structure of the scaffold is instrumental to entrap various cells types, including hematopoietic cells [53], and supports the deposition and presentation of extracellular matrix signals known to positively regulate MSC expansion [54], [55]. Based on the recent finding that typical stromal populations can form the niche to earlier, less adherent MSC progenitors which are removed with medium changes [20], it would be tempting to speculate that the stromal cell network generated within the scaffold offers the environmental cues required to support maintenance in culture of the earlier progenitors.

In the present study, a ceramic-based material has been used as a surface for initial adhesion and growth of MSC, in order to mimic some features of the mineralized trabeculae surrounding a marrow stromal tissue. It is likely that the use of materials of different composition, architecture and surface properties would provide different priming signals to marrow cells. Thus, the choice of the scaffold included in the perfusion chamber could represent a critical parameter of the system and at the same time an additional tool to dissect the role of specific factors in maintenance of MSC features. The 3D culture process critically requires the use of direct perfusion, initially in order to uniformly distribute cells throughout the scaffold pores [26] and later to efficiently nourish the cells down to the scaffold core. Moreover, the induced perfusion would also mimic the physiological role of interstitial fluid flow and associated mechanical shear in the bone environment [56]–[58]. Our previous study, though with animal derived BMSC and slightly different medium composition, has indicated the effect of continuous flow during culture in maintaining the presence of hematopoietic lineage cells [59]. Therefore, an experimental setup involving 3D cell cultures under static conditions or by perfusing pre-sorted CD45- cells from bone marrow preparations could identify the role of hematopoietic lineage cells in the maintenance of MSC functionality.

## Conclusions

In this work we have proposed an unprecedented paradigm for human MSC expansion, which – unlike most so far reported methods – does not rely on plastic adherence to initiate the culture. The described system relies on the in vitro establishment of a 3D stromal environment as a biomimetic niche supporting MSC growth while better preserving their functional properties. The complete elimination of the labour-extensive serial passaging in monolayer and the use of a perfusion-based bioreactor open the perspective of a streamlined, automated and controlled MSC expansion within closed systems, possibly addressing not only cell quality issues but also cost effectiveness and standardization of the manufacturing process for clinical and industrial implementation [60]. From a scientific perspective, the culture method offers the possibility to systematically investigate how different parameters (e.g., scaffold composition, architecture and functionalization, flow rate) regulate the phenotype, growth and function of the generated cell populations, and could be used as an engineered 3D model of the bone marrow stromal environment to study physiological interactions among multiple cell types. Finally, the approach may be extended to other stem cell systems, of interest in fundamental research, molecular medicine and cellular therapy.

## Supporting Information

Figure S1 **Anti-proliferative effect of MSC, expanded either by 3D-perfusion or 2D, on CD4+ activated cells.**
(TIF)

Figure S2 **QRT-PCR evaluation of gene expression for selected genes for the preparations (prep) derived from five different donors to validate the microarray data.** Legends: 3D represents the 3D-perfusion condition.
(TIF)

Table S1 **Biological processes correlated with MSC genes that were up-regulated at least two-folds in 3D-perfusion as compared to 2D cultures.** Terms are ordered according to their p-values.
(DOCX)

Table S2 **Biological processes correlated with MSC genes that were down-regulated at least two-folds in 3D-perfusion as compared to 2D cultures.** Terms are ordered according to their p-values.
(DOCX)

## References

[1] Uccelli A, Moretta L, Pistoia V (2008) Mesenchymal stem cells in health and disease. Nat Rev Immunol 8: 726–736 10.1038/nri2395 [doi] 19172693

[2] Almeida-Porada G, Porada CD, Tran N, Zanjani ED (2000) Cotransplantation of human stromal cell progenitors into preimmune fetal sheep results in early appearance of human donor cells in circulation and boosts cell levels in bone marrow at later time points after transplantation. Blood 95: 3620–3627. 10828053

[3] Pereira RF, Halford KW, O'Hara MD, Leeper DB, Sokolov BP, et al (1995) Cultured adherent cells from marrow can serve as long-lasting precursor cells for bone, cartilage, and lung in irradiated mice. Proc Natl Acad Sci U S A 92: 4857–4861. 7761413 10.1073/pnas.92.11.4857 PMC41806

[4] Orlic D, Kajstura J, Chimenti S, Jakoniuk I, Anderson SM, et al (2001) Bone marrow cells regenerate infarcted myocardium. Nature 410: 701–705 10.1038/35070587 [doi];35070587 [pii] 11287958

[5] Banfi A, Muraglia A, Dozin B, Mastrogiacomo M, Cancedda R, et al (2000) Proliferation kinetics and differentiation potential of ex vivo expanded human bone marrow stromal cells: Implications for their use in cell therapy. Exp Hematol 28: 707–715. S0301-472X(00)00160-0 [pii] 10880757 10.1016/s0301-472x(00)00160-0

[6] Banfi A, Bianchi G, Notaro R, Luzzatto L, Cancedda R, et al (2002) Replicative aging and gene expression in long-term cultures of human bone marrow stromal cells. Tissue Eng 8: 901–910 10.1089/107632702320934001 [doi] 12542936

[7] Augello A, Kurth TB, De BC (2010) Mesenchymal stem cells: a perspective from in vitro cultures to in vivo migration and niches. Eur Cell Mater 20: 121–133. vol020a11 [pii] 21249629 10.22203/ecm.v020a11

[8] da Silva ML, Caplan AI, Nardi NB (2008) In search of the in vivo identity of mesenchymal stem cells. Stem Cells 26: 2287–2299 2007–1122 [pii]; 10.1634/stemcells.2007-1122 [doi] 18566331

[9] Lai Y, Sun Y, Skinner CM, Son EL, Lu Z, et al (2010) Reconstitution of marrow-derived extracellular matrix ex vivo: a robust culture system for expanding large-scale highly functional human mesenchymal stem cells. Stem Cells Dev 19: 1095–1107 10.1089/scd.2009.0217 [doi] 19737070 PMC3128312

[10] Mendez-Ferrer S, Michurina TV, Ferraro F, Mazloom AR, Macarthur BD, et al (2010) Mesenchymal and haematopoietic stem cells form a unique bone marrow niche. Nature 466: 829–834 nature09262 [pii]; 10.1038/nature09262 [doi] 20703299 PMC3146551

[11] Scadden DT (2006) The stem-cell niche as an entity of action. Nature 441: 1075–1079 nature04957 [pii]; 10.1038/nature04957 [doi] 16810242

[12] Bara JJ, Richards RG, Alini M, Stoddart MJ (2014) Bone marrow-derived mesenchymal stem cells change phenotype following in vitro culture: Implications for basic research and the clinic. Stem Cells. 10.1002/stem.1649 [doi] 24449458

[13] Chen X, Xu H, Wan C, McCaigue M, Li G (2006) Bioreactor expansion of human adult bone marrow-derived mesenchymal stem cells. Stem Cells 24: 2052–2059 2005-0591 [pii]; 10.1634/stemcells.2005-0591 [doi] 16728560

[14] Frith JE, Thomson B, Genever PG (2010) Dynamic three-dimensional culture methods enhance mesenchymal stem cell properties and increase therapeutic potential. Tissue Eng Part C Methods 16: 735–749 10.1089/ten.TEC.2009.0432 [doi] 19811095

[15] Eibes G, dos SF, Andrade PZ, Boura JS, Abecasis MM, et al (2010) Maximizing the ex vivo expansion of human mesenchymal stem cells using a microcarrier-based stirred culture system. J Biotechnol 146: 194–197 S0168-1656(10)00101-X [pii]; 10.1016/j.jbiotec.2010.02.015 [doi] 20188771

[16] Yang Y, Rossi FM, Putnins EE (2007) Ex vivo expansion of rat bone marrow mesenchymal stromal cells on microcarrier beads in spin culture. Biomaterials 28: 3110–3120 S0142-9612(07)00237-2 [pii]; 10.1016/j.biomaterials.2007.03.015 [doi] 17433434

[17] Frauenschuh S, Reichmann E, Ibold Y, Goetz PM, Sittinger M, et al (2007) A microcarrier-based cultivation system for expansion of primary mesenchymal stem cells. Biotechnol Prog 23: 187–193 10.1021/bp060155w [doi] 17269687

[18] Diederichs S, Roker S, Marten D, Peterbauer A, Scheper T, et al (2009) Dynamic cultivation of human mesenchymal stem cells in a rotating bed bioreactor system based on the Z RP platform. Biotechnol Prog 25: 1762–1771 10.1002/btpr.258 [doi] 19795480

[19] Reichardt A, Polchow B, Shakibaei M, Henrich W, Hetzer R, et al (2013) Large scale expansion of human umbilical cord cells in a rotating bed system bioreactor for cardiovascular tissue engineering applications. Open Biomed Eng J 7: 50–61 10.2174/1874120701307010050 [doi];TOBEJ-7-50 [pii] 23847691 PMC3706833

[20] Di Maggio N, Mehrkens A, Papadimitropoulos A, Schaeren S, Heberer M, et al (2012) Fibroblast growth factor-2 maintains a niche-dependent population of self-renewing highly potent non-adherent mesenchymal progenitors through FGFR2c. Stem Cells 30: 1455–1464 10.1002/stem.1106 [doi] 22495904

[21] Eipers PG, Kale S, Taichman RS, Pipia GG, Swords NA, et al (2000) Bone marrow accessory cells regulate human bone precursor cell development. Exp Hematol 28: 815–825. S0301472X00001831 [pii] 10907643 10.1016/s0301-472x(00)00183-1

[22] Baksh D, Davies JE, Zandstra PW (2003) Adult human bone marrow-derived mesenchymal progenitor cells are capable of adhesion-independent survival and expansion. Exp Hematol 31: 723–732. S0301472X03001061 [pii] 12901978 10.1016/s0301-472x(03)00106-1

[23] Baksh D, Davies JE, Zandstra PW (2005) Soluble factor cross-talk between human bone marrow-derived hematopoietic and mesenchymal cells enhances in vitro CFU-F and CFU-O growth and reveals heterogeneity in the mesenchymal progenitor cell compartment. Blood 106: 3012–3019 2005-01-0433 [pii]; 10.1182/blood-2005-01-0433 [doi] 16030193

[24] Braccini A, Wendt D, Jaquiery C, Jakob M, Heberer M, et al (2005) Three-dimensional perfusion culture of human bone marrow cells and generation of osteoinductive grafts. Stem Cells 23: 1066–1072 2005-0002 [pii]; 10.1634/stemcells.2005-0002 [doi] 16002780

[25] Di Maggio N, Piccinini E, Jaworski M, Trumpp A, Wendt DJ, et al (2011) Toward modeling the bone marrow niche using scaffold-based 3D culture systems. Biomaterials 32: 321–329 S0142-9612(10)01238-X [pii]; 10.1016/j.biomaterials.2010.09.041 [doi] 20952054

[26] Wendt D, Marsano A, Jakob M, Heberer M, Martin I (2003) Oscillating perfusion of cell suspensions through three-dimensional scaffolds enhances cell seeding efficiency and uniformity. Biotechnol Bioeng 84: 205–214 10.1002/bit.10759 [doi] 12966577

[27] Merico D, Isserlin R, Stueker O, Emili A, Bader GD (2010) Enrichment map: a network-based method for gene-set enrichment visualization and interpretation. PLoS One 5: e13984 10.1371/journal.pone.0013984 [doi] 21085593 PMC2981572

[28] Shannon P, Markiel A, Ozier O, Baliga NS, Wang JT, et al (2003) Cytoscape: a software environment for integrated models of biomolecular interaction networks. Genome Res 13: 2498–2504 10.1101/gr.1239303 [doi];13/11/2498 [pii] 14597658 PMC403769

[29] Mauney JR, Jaquiery C, Volloch V, Heberer M, Martin I, et al (2005) In vitro and in vivo evaluation of differentially demineralized cancellous bone scaffolds combined with human bone marrow stromal cells for tissue engineering. Biomaterials 26: 3173–3185 S0142-9612(04)00769-0 [pii]; 10.1016/j.biomaterials.2004.08.020 [doi] 15603812

[30] Barbero A, Ploegert S, Heberer M, Martin I (2003) Plasticity of clonal populations of dedifferentiated adult human articular chondrocytes. Arthritis Rheum 48: 1315–1325 10.1002/art.10950 [doi] 12746904

[31] Bocelli-Tyndall C, Bracci L, Schaeren S, Feder-Mengus C, Barbero A, et al (2009) Human bone marrow mesenchymal stem cells and chondrocytes promote and/or suppress the in vitro proliferation of lymphocytes stimulated by interleukins 2, 7 and 15. Ann Rheum Dis 68: 1352–1359 ard.2008.094003 [pii]; 10.1136/ard.2008.094003 [doi] 18647856

[32] Pittenger MF, Mackay AM, Beck SC, Jaiswal RK, Douglas R, et al (1999) Multilineage potential of adult human mesenchymal stem cells. Science 284: 143–147. 10102814 10.1126/science.284.5411.143

[33] Harichandan A, Buhring HJ (2011) Prospective isolation of human MSC. Best Pract Res Clin Haematol 24: 25–36 S1521-6926(11)00002-8 [pii]; 10.1016/j.beha.2011.01.001 [doi] 21396590

[34] Russell KC, Phinney DG, Lacey MR, Barrilleaux BL, Meyertholen KE, et al (2010) In vitro high-capacity assay to quantify the clonal heterogeneity in trilineage potential of mesenchymal stem cells reveals a complex hierarchy of lineage commitment. Stem Cells 28: 788–798 10.1002/stem.312 [doi] 20127798

[35] Sorrentino A, Ferracin M, Castelli G, Biffoni M, Tomaselli G, et al (2008) Isolation and characterization of CD146+ multipotent mesenchymal stromal cells. Exp Hematol 36: 1035–1046 S0301-472X(08)00118-5 [pii]; 10.1016/j.exphem.2008.03.004 [doi] 18504067

[36] Anjos-Afonso F, Bonnet D (2007) Nonhematopoietic/endothelial SSEA-1+ cells define the most primitive progenitors in the adult murine bone marrow mesenchymal compartment. Blood 109: 1298–1306 blood-2006-06-030551 [pii]; 10.1182/blood-2006-06-030551 [doi] 17003364

[37] Sacchetti B, Funari A, Michienzi S, Di CS, Piersanti S, et al (2007) Self-renewing osteoprogenitors in bone marrow sinusoids can organize a hematopoietic microenvironment. Cell 131: 324–336 S0092-8674(07)01087-2 [pii]; 10.1016/j.cell.2007.08.025 [doi] 17956733

[38] Raychaudhuri S, Stuart JM, Altman RB (2000) Principal components analysis to summarize microarray experiments: application to sporulation time series. Pac Symp Biocomput 455–466. 10902193 10.1142/9789814447331_0043 PMC2669932

[39] Muller FJ, Laurent LC, Kostka D, Ulitsky I, Williams R, et al (2008) Regulatory networks define phenotypic classes of human stem cell lines. Nature 455: 401–405 nature07213 [pii]; 10.1038/nature07213 [doi] 18724358 PMC2637443

[40] Bianco P, Robey PG, Simmons PJ (2008) Mesenchymal stem cells: revisiting history, concepts, and assays. Cell Stem Cell 2: 313–319 S1934-5909(08)00114-8 [pii]; 10.1016/j.stem.2008.03.002 [doi] 18397751 PMC2613570

[41] Dominici M, Le BK, Mueller I, Slaper-Cortenbach I, Marini F, et al (2006) Minimal criteria for defining multipotent mesenchymal stromal cells. The International Society for Cellular Therapy position statement. Cytotherapy 8: 315–317 Q2183N8UT042W62H [pii]; 10.1080/14653240600855905 [doi] 16923606

[42] Bocelli-Tyndall C, Zajac P, Di Maggio N, Trella E, Benvenuto F, et al (2010) Fibroblast growth factor 2 and platelet-derived growth factor, but not platelet lysate, induce proliferation-dependent, functional class II major histocompatibility complex antigen in human mesenchymal stem cells. Arthritis Rheum 62: 3815–3825 10.1002/art.27736 [doi] 20824797

[43] Tarte K, Gaillard J, Lataillade JJ, Fouillard L, Becker M, et al (2010) Clinical-grade production of human mesenchymal stromal cells: occurrence of aneuploidy without transformation. Blood 115: 1549–1553 blood-2009-05-219907 [pii]; 10.1182/blood-2009-05-219907 [doi] 20032501

[44] Muraglia A, Cancedda R, Quarto R (2000) Clonal mesenchymal progenitors from human bone marrow differentiate in vitro according to a hierarchical model. J Cell Sci 113 (Pt 7): 1161–1166. 10704367 10.1242/jcs.113.7.1161

[45] Zhu J, Shimizu E, Zhang X, Partridge NC, Qin L (2011) EGFR signaling suppresses osteoblast differentiation and inhibits expression of master osteoblastic transcription factors Runx2 and Osterix. J Cell Biochem 112: 1749–1760 10.1002/jcb.23094 [doi] 21381079 PMC3111753

[46] Pevsner-Fischer M, Morad V, Cohen-Sfady M, Rousso-Noori L, Zanin-Zhorov A, et al (2007) Toll-like receptors and their ligands control mesenchymal stem cell functions. Blood 109: 1422–1432 blood-2006-06-028704 [pii]; 10.1182/blood-2006-06-028704 [doi] 17038530

[47] Fehrer C, Brunauer R, Laschober G, Unterluggauer H, Reitinger S, et al (2007) Reduced oxygen tension attenuates differentiation capacity of human mesenchymal stem cells and prolongs their lifespan. Aging Cell 6: 745–757 ACE336 [pii]; 10.1111/j.1474-9726.2007.00336.x [doi] 17925003

[48] Estrada JC, Albo C, Benguria A, Dopazo A, Lopez-Romero P, et al (2012) Culture of human mesenchymal stem cells at low oxygen tension improves growth and genetic stability by activating glycolysis. Cell Death Differ 19: 743–755 cdd2011172 [pii]; 10.1038/cdd.2011.172 [doi] 22139129 PMC3321628

[49] Pedersen JA, Swartz MA (2005) Mechanobiology in the third dimension. Ann Biomed Eng 33: 1469–1490 10.1007/s10439-005-8159-4 [doi] 16341917

[50] Trappmann B, Gautrot JE, Connelly JT, Strange DG, Li Y, et al (2012) Extracellular-matrix tethering regulates stem-cell fate. Nat Mater 11: 642–649 nmat3339 [pii]; 10.1038/nmat3339 [doi] 22635042

[51] Li B, Moshfegh C, Lin Z, Albuschies J, Vogel V (2013) Mesenchymal stem cells exploit extracellular matrix as mechanotransducer. Sci Rep 3: 2425 srep02425 [pii]; 10.1038/srep02425 [doi] 23939587 PMC3741624

[52] Cukierman E, Pankov R, Yamada KM (2002) Cell interactions with three-dimensional matrices. Curr Opin Cell Biol 14: 633–639. S0955067402003642 [pii] 12231360 10.1016/s0955-0674(02)00364-2

[53] Zhang ZL, Tong J, Lu RN, Scutt AM, Goltzman D, et al (2009) Therapeutic potential of non-adherent BM-derived mesenchymal stem cells in tissue regeneration. Bone Marrow Transplant 43: 69–81 bmt2008260 [pii]; 10.1038/bmt.2008.260 [doi] 18711348

[54] Chen XD, Dusevich V, Feng JQ, Manolagas SC, Jilka RL (2007) Extracellular matrix made by bone marrow cells facilitates expansion of marrow-derived mesenchymal progenitor cells and prevents their differentiation into osteoblasts. J Bone Miner Res 22: 1943–1956 10.1359/jbmr.070725 [doi] 17680726

[55] Prewitz MC, Seib FP, von BM, Friedrichs J, Stissel A, et al (2013) Tightly anchored tissue-mimetic matrices as instructive stem cell microenvironments. Nat Methods 10: 788–794 nmeth.2523 [pii]; 10.1038/nmeth.2523 [doi] 23793238

[56] Hillsley MV, Frangos JA (1994) Bone tissue engineering: the role of interstitial fluid flow. Biotechnol Bioeng 43: 573–581 10.1002/bit.260430706 [doi] 11540959

[57] Owan I, Burr DB, Turner CH, Qiu J, Tu Y, et al (1997) Mechanotransduction in bone: osteoblasts are more responsive to fluid forces than mechanical strain. Am J Physiol 273: C810–C815. 9316399 10.1152/ajpcell.1997.273.3.C810

[58] You J, Yellowley CE, Donahue HJ, Zhang Y, Chen Q, et al (2000) Substrate deformation levels associated with routine physical activity are less stimulatory to bone cells relative to loading-induced oscillatory fluid flow. J Biomech Eng 122: 387–393. 11036562 10.1115/1.1287161

[59] Scaglione S, Braccini A, Wendt D, Jaquiery C, Beltrame F, et al (2006) Engineering of osteoinductive grafts by isolation and expansion of ovine bone marrow stromal cells directly on 3D ceramic scaffolds. Biotechnol Bioeng 93: 181–187 10.1002/bit.20677 [doi] 16245346

[60] Martin I, Simmons PJ, Williams DF (2014) Manufacturing Challenges in Regenerative Medicine. Sci. Transl. Med. 6, 232fs16 10.1126/scitranslmed.3008558 24739757

