# Supplementary figures and images for: Expansion of Human Mesenchymal Stromal Cells from Fresh Bone Marrow in a 3D Scaffold-Based System under Direct Perfusion
Source: PLoS One. 2014 Jul 14;9(7):e102359. doi: 10.1371/journal.pone.0102359 (PMC4096512; doi:10.1371/journal.pone.0102359)

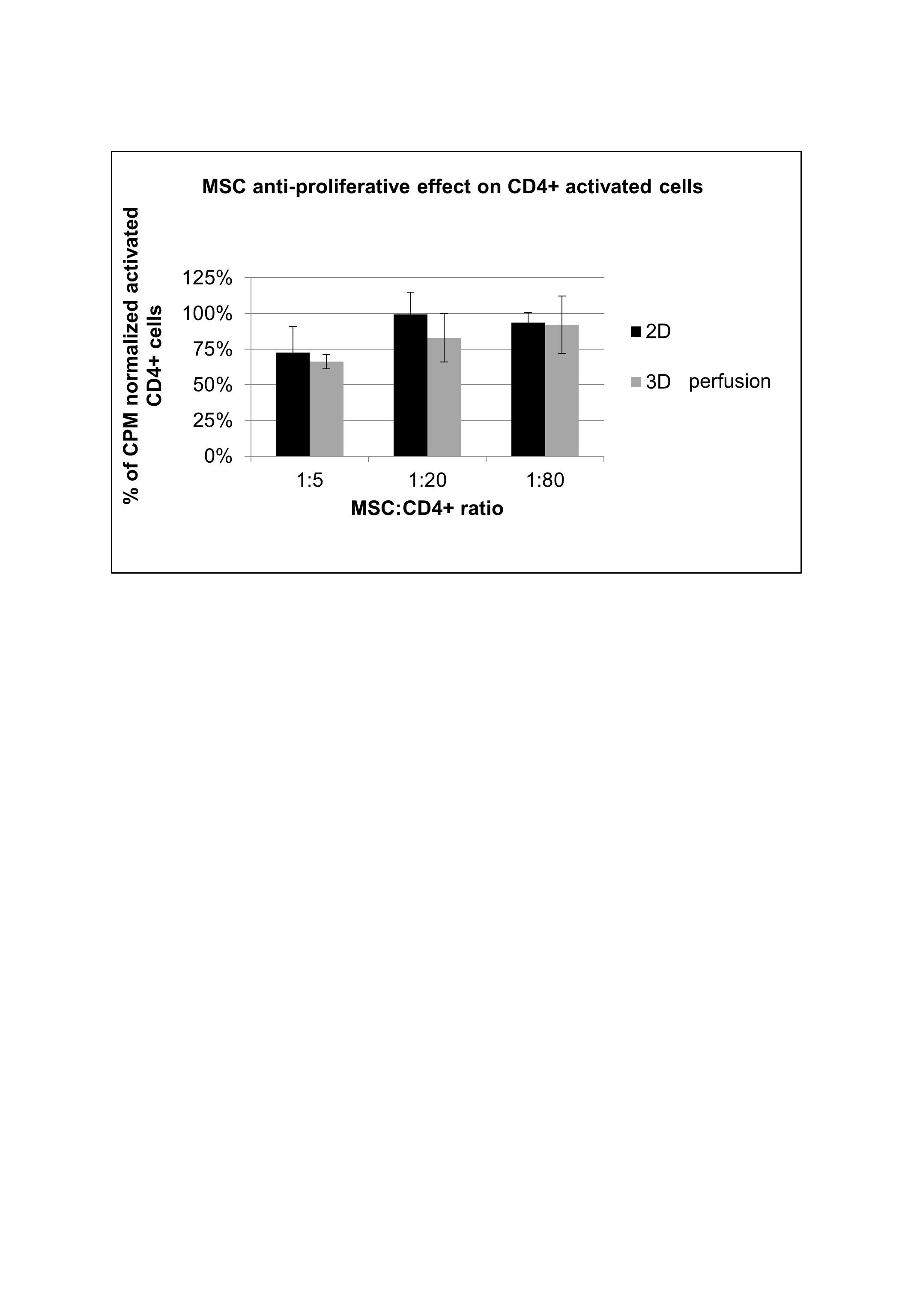

Supplement: Figure S1 — Anti-proliferative effect of MSC, expanded either by 3D-perfusion or 2D, on CD4+ activated cells. (TIF) [file pone.0102359.s001.tif]

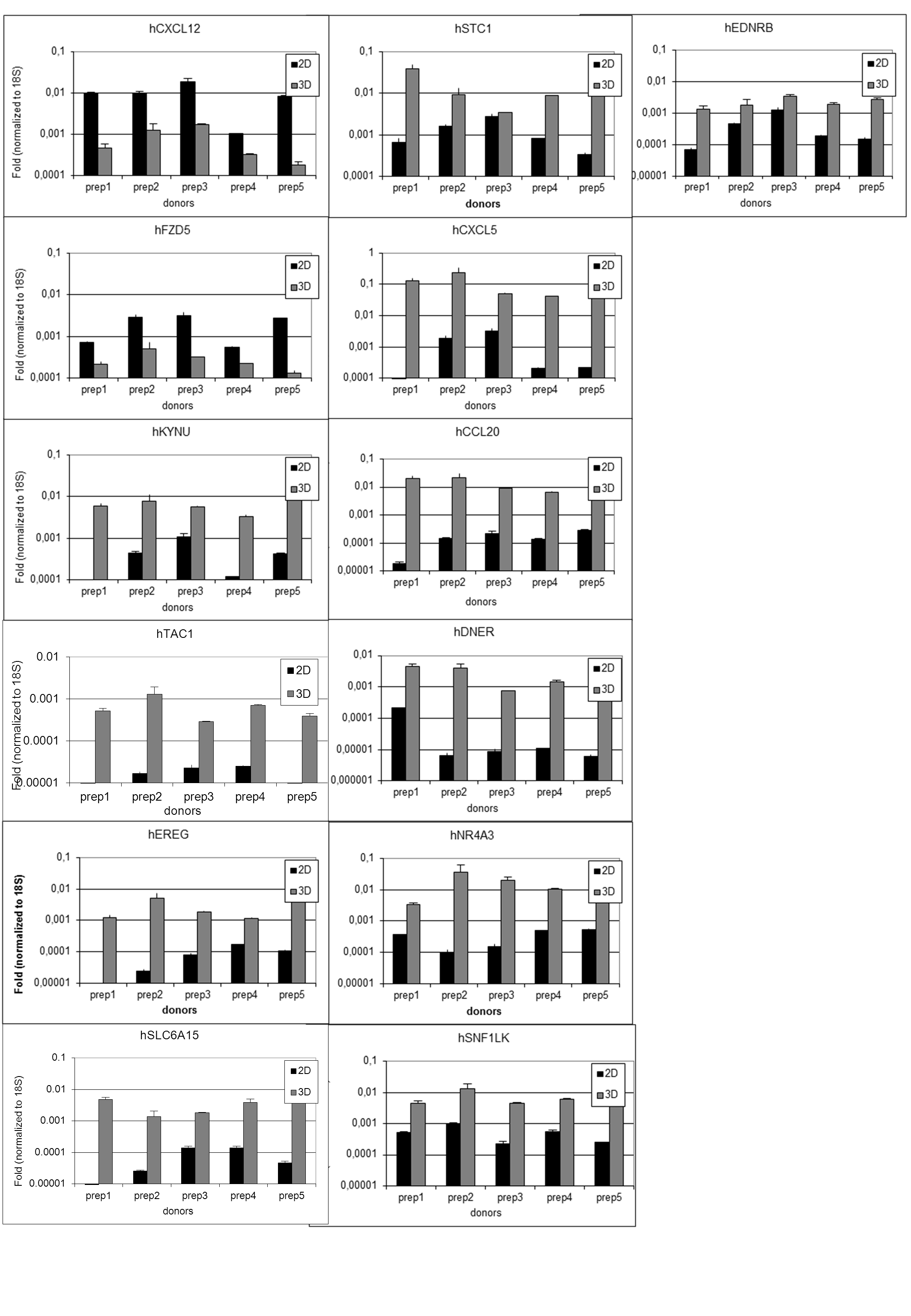

Supplement: Figure S2 — QRT-PCR evaluation of gene expression for selected genes for the preparations (prep) derived from five different donors to validate the microarray data. Legends: 3D represents the 3D-perfusion condition. (TIF) [file pone.0102359.s002.tif]
