# Supplementary material for: Expansion of Human Mesenchymal Stromal Cells from Fresh Bone Marrow in a 3D Scaffold-Based System under Direct Perfusion
Source: PLoS One. 2014 Jul 14;9(7):e102359. doi: 10.1371/journal.pone.0102359 (PMC4096512; doi:10.1371/journal.pone.0102359)
Supplement: Table S1 — Biological processes correlated with MSC genes that were up-regulated at least two-folds in 3D-perfusion as compared to 2D cultures. Terms are ordered according to their p-values. (DOCX) [file pone.0102359.s003.docx]

| **TERM** | **Input genes in GO terms (%)** | **P-Value** | **TERM** | **Input genes in GO terms (%)** | **P-Value** |
| --- | --- | --- | --- | --- | --- |
| hexose metabolic process | 7,0 | 2,4E-8 | response to wounding | 6,2 | 7,6E-4 |
| monosaccharide metabolic process | 7,0 | 1,8E-7 | locomotory behavior | 5,0 | 7,6E-4 |
| extracellular space | 11,6 | 6,0E-7 | response to organic substance | 2,9 | 7,9E-4 |
| cytokine activity | 6,2 | 6,1E-7 | positive regulation of response to stimulus | 7,9 | 8,1E-4 |
| glucose metabolic process | 5,4 | 2,7E-6 | cytosol | 3,7 | 9,8E-4 |
| extracellular region part | 3,3 | 2,7E-6 | positive regulation of defense response | 9,5 | 1,1E-3 |
| chemokine activity | 2,5 | 3,9E-6 | response to hypoxia | 3,7 | 1,3E-3 |
| fructose metabolic process | 3,3 | 4,4E-6 | response to oxygen levels | 2,1 | 1,3E-3 |
| chemokine receptor binding | 13,2 | 6,4E-6 | behavior | 8,3 | 1,3E-3 |
| taxis | 5,0 | 2,1E-5 | defense response | 7,0 | 1,5E-3 |
| chemotaxis | 5,0 | 2,1E-5 | NOD-like receptor signaling pathway | 13,6 | 1,6E-3 |
| regulation of cell proliferation | 5,4 | 2,7E-4 | negative regulation of apoptosis | 2,9 | 1,7E-3 |
| magnesium ion binding | 7,4 | 2,8E-4 |  |  |  |
| positive regulation of multicellular organismal process | 10,7 | 2,8E-4 |  |  |  |
| inflammatory response | 6,2 | 3,0E-4 |  |  |  |
| positive regulation of cytokine production | 3,3 | 3,8E-4 |  |  |  |
| Fructose and mannose metabolism | 2,5 | 6,3E-4 |  |  |  |
| Cytokine-cytokine receptor interaction | 5,4 | 6,5E-4 |  |  |  |
