# Supplementary material for: Expansion of Human Mesenchymal Stromal Cells from Fresh Bone Marrow in a 3D Scaffold-Based System under Direct Perfusion
Source: PLoS One. 2014 Jul 14;9(7):e102359. doi: 10.1371/journal.pone.0102359 (PMC4096512; doi:10.1371/journal.pone.0102359)
Supplement: Table S2 — Biological processes correlated with MSC genes that were down-regulated at least two-folds in 3D-perfusion as compared to 2D cultures. Terms are ordered according to their p-values. (DOCX) [file pone.0102359.s004.docx]

| **TERM** | **Input genes in GO terms (%)** | **P-Value** | **TERM** | **Input genes in GO terms (%)** | **P-Value** |
| --- | --- | --- | --- | --- | --- |
| Actin binding cytoskeletal protein | 9.9 | 4.5E-10 | regulation of cell growth | 4.5 | 8.9E-04 |
| cytoskeletal protein binding | 11.3 | 2.3E-08 | organophosphate metabolic process | 4.5 | 9.2E-04 |
| Cytoskeletal protein | 12.6 | 7.5E-08 | Signaling by BMP | 1.8 | 1.0E-03 |
| actin cytoskeleton | 8.1 | 2.0E-07 | adherens junction | 4.1 | 1.5E-03 |
| actin binding | 8.1 | 7.8E-07 | transmembrane receptor protein serine/threonine kinase signaling pathway | 3.2 | 1.9E-03 |
| ossification | 4.1 | 1.1E-04 | basolateral plasma membrane | 4.5 | 2.2E-03 |
| bone development | 4.1 | 1.8E-04 | Sphingolipid metabolism | 2.3 | 2.4E-03 |
| contractile fiber part | 4.1 | 1.8E-04 | Extracellular matrix | 5.4 | 2.9E-03 |
| extracellular region | 20.3 | 1.9E-04 | anchoring junction | 4.1 | 2.9E-03 |
| BMP signaling pathway | 2.7 | 1.9E-04 | extracellular matrix | 5.9 | 2.9E-03 |
| insulin-like growth factor binding | 2.3 | 2.8E-04 | cell adhesion | 8.6 | 3.0E-03 |
| contractile fiber | 4.1 | 2.9E-04 | Cell adhesion molecule | 5.4 | 3.0E-03 |
| Non-motor actin binding protein | 4.1 | 4.3E-04 | biological adhesion | 8.6 | 3.0E-03 |
| extracellular region part | 12.2 | 5.0E-04 | cytoskeleton organization | 6.3 | 3.4E-03 |
| phospholipid metabolic process | 4.5 | 6.3E-04 |  |  |  |
| regulation of cellular component size | 5.4 | 6.6E-04 |  |  |  |
